# Korean Mothers Attune the Frequency and Acoustic Saliency of Sound Symbolic Words to the Linguistic Maturity of Their Children

**DOI:** 10.3389/fpsyg.2018.02225

**Published:** 2018-12-18

**Authors:** Jinyoung Jo, Eon-Suk Ko

**Affiliations:** ^1^Department of English Language and Literature, Seoul National University, Seoul, South Korea; ^2^Department of English Language and Literature, Chosun University, Gwangju, South Korea

**Keywords:** sound symbolism, child-directed speech, Korean mothers, developmental changes, mother-child interaction, CHILDES, phonological iconicity

## Abstract

The present study investigates Korean mothers’ use of sound symbolism, in particular expressive lengthening and ideophones, in their speech directed to their children. Specifically, we explore whether the frequency and acoustic saliency of sound symbolic words are modulated by the maturity of children’s linguistic ability. A total of 36 infant-mother dyads, 12 each belonging to the three groups of preverbal (*M* = 8-month-old), early speech (*M* = 13-month-old), and multiword (*M* = 27-month-old) stage, were recorded in a 40-min free-play session. The results were consistent with the findings in previous research that the ratio of sound symbolic words in mothers’ speech decreases with child age and that they are acoustically more salient than conventional words in duration and pitch measures. We additionally found that mothers weaken the prominence for ideophones for older children in mean pitch, suggesting that such prominence of these iconic words might bootstrap infants’ word learning especially when they are younger. Interestingly, however, we found that mothers maintain the acoustic saliency of expressive lengthening consistently across children’s ages in all acoustic measures. There is some indication that children at age 2 are not likely to have mastered the fine details of scalar properties in certain words. Thus, it could be that they still benefit from the enhanced prosody of expressive lengthening in learning the semantic attributes of scalar adjectives, and, accordingly, mothers continue to provide redundant acoustic cues longer for expressive lengthening than ideophones.

## Introduction

### Sound Symbolism and Its Psychological Reality

Arbitrariness between linguistic form and meaning has long been considered one of the important design features of human language (de Saussure, 1916; Hockett, 1973). It has traditionally been assumed that sound symbolism, the non-arbitrary relationship between sound and meaning of a word (e.g., *bang*), is a marginal phenomenon in language. There is, however, a growing body of research investigating non-arbitrary aspects of linguistic form and meaning (e.g., onomatopoeia), especially with regard to three central areas in linguistics, i.e., language evolution(Ramachandran and Hubbard, 2001), language learning (Imai et al., 2008, 2015; Imai and Kita, 2014; Perry et al., 2017), and language processing (Thompson, 2011).

Sound symbolism, or phonological iconicity, is found more abundantly in natural languages than one might assume (Perniss et al., 2010). For example, many languages have a distinct grammatical category of sound symbolic words referred to as mimetics, ideophones, or expressives. Such inventories exist in many East Asian languages (for Korean see Lee, 1992; for Japanese see Hamano, 1998), most sub-Saharan languages (Childs, 1994), a number of Southeast Asian languages (Diffloth, 1972; Watson, 2001), and some South American languages (Derbyshire and Pullum, 1986), just to name a few. Sound symbolic words in these languages not only depict various sensory imageries (beyond acoustic perception as in onomatopoeia), but also describe affective states and manners of motion. Productivity of sound symbolic words has been shown in naming proper nouns (Kawahara et al., 2018).

Non-arbitrary sound-meaning correspondence also exists in languages that do not have a grammatically defined category of sound symbolic words. For example, several cases of phonaesthemes are found in English in which a consonant cluster at the beginning or the end of some words represents specific meanings; /gl/ in English as in *glitter*, *glare*, *glow*, *gleam*, etc., has meanings related to vision and light and similarly, an /sn/ sequence containing a nasal sound occurs in words related to nose as in *snore, sniff*, and *snob* “someone with their nose in the air” (Schmidtke et al., 2014). Also, there is evidence showing that speakers actively use such regular phonological mappings found in phonaesthemes in production and perception of neologisms (Hutchins, 1998; Magnus, 2000). Thus, the principle of arbitrary relationship between the sound of a word and its lexical meaning, as assumed in traditional linguistics, seems to be too strong.

The psychological reality of sound symbolism has been widely documented (e.g., Köhler, 1929; Sapir, 1929; Ohala, 1994; Westbury, 2005; Bremner et al., 2013), especially in studies investigating sound-shape or sound-size correspondences. In Köhler’s (1929) pioneering work, when speakers of different languages were presented with two novel objects, one rounded and the other spikey, they judged *maluma* to be a better name for the rounded object and *takete* for the spikey object. The finding was replicated in several other studies (Westbury, 2005; Bremner et al., 2013), suggesting that one has the intuition that certain sound-shape correspondences are better than others. Also, Sapir (1929) demonstrated that [i] was judged to be more appropriate as a label for small objects and [a] for larger objects; similar effects were found across several languages (Ultan, 1978; Ohala, 1994).

Importantly, young children also exhibit sensitivity to sound symbolism as reported in many previous studies (Maurer et al., 2006; Imai et al., 2008; Peña et al., 2011). Maurer et al. (2006) found that Canadian 2.5-year-olds were able to map rounded vowels to rounded shapes and unrounded vowels to unrounded shapes, suggesting that they are sensitive to the sound-shape mapping shown in Köhler (1929). Sensitivity to systematic sound-size correspondences (Sapir, 1929) has also been observed. Using two-way preferential looking paradigm, Peña et al. (2011) found that 4-month-old infants learning Spanish were able to match [i, e] with small objects and [o, a] with large objects, as indicated by the fact that the infants looked at the sound-symbolically congruent stimuli longer than at the mismatching ones. In the domain of manner of motion, Imai et al. (2008) showed that when asked to choose one of two video clips that a given sound symbolic verb referred to, Japanese 25-month-olds selected the one that sound-symbolically matched the word. These findings suggest that children’s sensitivity to sound symbolism is cross-linguistically observed in various domains of sound-meaning mapping, even in very young infants.

### The Sound Symbolism Bootstrap Hypothesis of Word Learning

It has been traditionally assumed that iconic symbol-to-referent mappings facilitate young children’s symbol acquisition (Piaget, 1962; Werner and Kaplan, 1963). In particular, the close resemblance between form and meaning in sound symbolic expressions has been claimed to make it easier for children to tackle the task of word learning (Farrar, 1883; Berko-Gleason, 2005; Imai and Kita, 2014) and recent experimental research has in fact shown that children use their sensitivity to sound symbolism in word learning (Imai et al., 2015). According to Imai and Kita (2014), sound symbolism bootstraps lexical development because infants are endowed with an innate multimodal mapping ability (e.g., sound and vision). When infants are provided with an auditory and a visual stimulus, for example, they spontaneously integrate the two stimuli. Crucially, this process is facilitated if there is an iconic relationship between the two stimuli, as evidenced by infants’ different neural responses to sound-symbolically matching and mismatching stimuli (Asano et al., 2015). This may ultimately help infants realize that speech sounds refer to entities in the world, thus promoting the acquisition of word meaning. But see Orlansky and Bonvillian (1984) and Woodward et al. (1994) for findings that show no clear advantage for iconicity in symbol learning.

Meanwhile, other studies provide an input-based account of why sound symbolic words are easier to learn (Kunnari, 2002; Laing et al., 2016). Particularly relevant to the present study is Laing et al.’s (2016) finding that onomatopoeic words in child-directed speech were more salient than conventional words in a number of acoustic properties such as mean pitch, pitch range, and word duration. They propose that such acoustic saliency may contribute to the early acquisition of onomatopoeic words as documented in previous studies (Kern, 2010; Menn and Vihman, 2011). As will be explained shortly, the current study extends Laing et al.’s study by investigating the effect of child age on the perceptual saliency of sound symbolic words in the input.

Presumably due to infants’ innate bias toward sound symbolic words and their acoustic saliency in the input, sound symbolism is a common feature of child speech. Kern (2010) reported, for instance, that onomatopoeia accounts for more than a third of French infants’ lexicon between the ages of 0;8 and 1;4. In a cross-linguistic study, Menn and Vihman (2011) found that onomatopoeia constitutes 20% of the infants’ first five words in ten different languages. Moreover, younger children produce sound symbolic words more often than older children (Kita et al., 2010). This is in parallel with mothers’ more frequent use of those words with younger children (Kauschke and Klann-delius, 2007).

A prediction following from the claim that sound symbolism facilitates word learning is that mother’s use of sound symbolism will be conditioned by the maturity of the child’s ability to associate linguistic form with meaning. Studies have indeed shown that mothers use sound symbolic words more frequently when talking to very young infants and the frequency of these forms decreases as the child’s language ability develops (Kauschke and Klann-delius, 2007; Saji and Imai, 2013; Perlman et al., 2017; Perry et al., 2017). In Saji and Imai (2013), for example, when Japanese mothers were asked to describe action events presented in videos, they used fewer sound symbolic words when speaking to 3-year-olds than to 2-year-olds, and still fewer to adults. Similarly, it was found in Kauschke and Klann-delius (2007) that “personal-social words” in the mothers’ speech, which included onomatopoeic words, decreased significantly across the four sampling points, i.e., 1;1, 1;3, 1;9, and 3;0 years of age. In addition, Perry et al. (2017) and Perlman et al. (2017) found that children use iconic words more frequently earlier in language learning but the proportion of these words decreases over time. They also found that such a pattern is mirrored in the speech of their mothers. Thus, the literature suggests that mothers’ use of sound symbolic words generally decreases as children’s linguistic ability develops (see Fernald and Morikawa, 1993 for slightly different results); onomatopoeic words showed the highest frequency in the intermediate age group (i.e., 12-month-olds) compared to the younger (i.e., 6-month-olds) or the older (i.e., 19-month-olds).

### Sound Symbolism in Korean: Ideophones and Expressive Lengthening

Korean has a large number of sound symbolic words with an iconic relationship between linguistic form and lexical meaning. A subcategory of sound symbolism in Korean is the lexical class of *ideophones*. Ideophonic words in Korean depict a wide range of sensory experiences and have traditionally been categorized into two semantic classes, i.e., *
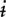
is*ə*ŋə* that imitates auditory experiences (e.g., *jaoŋ* “meow”) and *
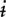
it𝜀ə* that imitates non-auditory information such as visual (e.g., *tuŋk
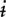
ltuŋk
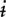
l* “round”), tactile (e.g., *k’ac^h^ilk’ac^h^il* “rough”), and psychological experiences (e.g., *təlk^h^ək* “unexpectedly”) as well as bodily movements (e.g., *k’aŋc^h^oŋk’aŋc^h^oŋ* “hopping”). Ideophones in Korean are subject to phonotactic restrictions that are not observed in non-iconic conventional words, in line with the cross-linguistic tendency of iconic words to have characteristic phonological features that distinguish them from ordinary vocabulary. For instance, they are among the restricted subsets of Korean lexicon that exhibit vowel harmony, though the degree to which the harmony pattern is observed varies with the degree of iconicity of the word (Kwon, 2018).

Another type of sound symbolism in Korean is *expressive lengthening*. The phenomenon of expressive lengthening is realized by extra elongation of a vowel as indicated by double colons (e.g., *k^h^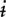::n* “huge,” *ə::mc^h^əŋ* “greatly”), and leads to augmentation and intensification of the scalar properties of the lexical meaning. It has been noted that many words in Korean, adjectives and adverbs in particular, undergo expressive lengthening and that the locus of lengthening is systematically modulated by the semantics of the word (Ko, 2017). We assume that not only segmental properties of sound symbolism (Maurer et al., 2006; Peña et al., 2011) but also durational differences (Perniss and Vigliocco, 2014) may be used to express variable size, length, degree, etc. in child-directed speech. Note that there is a phonological constraint on the application of expressive lengthening in English, allowing only monosyllables to undergo such lengthening (e.g., *hu::ge*, *fa::st*) whereas only semantic constraints are in operation in Korean. This difference thus makes the presence of this phenomenon in Korean spontaneous speech more predominant than in English.

According to Bae and Park (2012), the ratio of ideophonic words in Korean child-directed speech is reported to be particularly high (2.24%) compared to English (0.32%) or even to Japanese (1.01%). They also found that this distributional pattern is mirrored in the child’s speech: Korean-learning children produced a higher ratio of ideophones (2.73%) than those learning English (1.10%) or Japanese (2.15%). Moreover, as pointed out in Kwon (2018), Korean ideophones are typically produced as fully reduplicated forms. Considering that reduplication facilitates lexical learning (Ota and Skarabela, 2016) as well as early word segmentation (Ota, 2018), Korean mothers’ use of ideophones as reduplicated forms might assist children’s language learning.

### The Present Study

In this paper, we investigate Korean mothers’ use of sound symbolism, i.e., expressive lengthening and ideophones, in their speech directed to children. Specifically, we explore how the frequency and acoustic saliency of sound symbolic words are modulated by the maturity of children’s linguistic ability. Given that Korean has a rich inventory of sound symbolic words and that the frequency of these words is relatively higher than in other languages, Korean could be a potentially advantageous testing ground for investigating the use of sound symbolism in child-directed speech (CDS).

In addition to ideophones and expressive lengthening, we also examined the use of word play in child-directed speech, although this is not exactly categorized as sound symbolism. Word play includes different types of playful vocalizations, some cases of which are non-sense words produced (often without any specific meaning) mainly to gain the child’s attention and to amuse them. This category corresponds to what Fernald and Morikawa (1993) called “non-sense sounds used playfully but not attributed to a target object” (p. 643), which was found to be used more frequently in speech directed to younger infants.

We first investigate the frequency of words containing one of the three categories described above, i.e., expressive lengthening, ideophones, and word play, in mothers’ speech directed to children of different developmental stages. Considering that younger children who are still acquiring the ability to associate linguistic form with meaning are more likely to benefit from the inherent correspondence between sound and meaning found in sound symbolism and that mothers fine-tune their speech to children’s changing linguistic ability, we expect that the frequency of these forms in CDS will decrease as children’s linguistic ability develops.

We further investigate prosodic saliency of expressive lengthening and ideophonic words measured by word duration, mean pitch, and pitch range to examine if they were more acoustically prominent than non-iconic words, and if such saliency changes with child age. We reasoned that the acoustic saliency of sound symbolic words as reported in Laing et al. (2016) might also be modulated by children’s linguistic ability, as redundant cues present in enhanced acoustic saliency to signal the correspondence between linguistic form and meaning can be particularly helpful to younger infants. Therefore, our study extends Laing et al.’s findings in at least two ways. First, we test the prosodic saliency of a broader range of sound symbolic words. While Laing et al. focused on onomatopoeic words that imitate auditory experiences, the data investigated in the present study consist of ideophones depicting perceptual experiences in various modalities and words that underwent expressive lengthening. Second, we examined the effect of child age on the acoustic saliency of sound symbolic words with three groups of children at the preverbal (8 months), early speech (13 months), and multiword (27 months) developmental stages. Note that the infant participants in Laing et al.’s study were 8-month-old, which corresponds to the age of the youngest group in our study.

In sum, the present study investigates the following two questions with child-directed spontaneous speech elicited from Korean mothers. First, do Korean mothers rely less on the sound-meaning correspondence over the course of their children’s linguistic development? If so, we would expect to find a decrease in the proportion of the sound symbolic words in CDS with child age. Second, are sound symbolic words in Korean acoustically more salient than conventional words, and, further, does the level of saliency decrease as a function of child age? The first question, which addresses the frequency of sound symbolic words in CDS, is geared toward testing findings reported in other languages. The second question, regarding the interaction between age and acoustic measures, however, investigates an issue that has not yet been actively studied. The novel elements in the design of the current study should advance our understanding of the role of sound symbolism in word learning.

## Materials and Methods

### Creation of the Corpus

A total of 36 infant-mother dyads participated in the study, 12 each belonging to the three age groups, i.e., Ages 0, 1, and 2. The mean age of mothers was 33.8 (range = 28 to 41). Among the 36 mothers, 2 had a high school degree, 23 had a college degree, and 11 were pursuing or had obtained an advanced degree. We screened the infants’ language development at the time of recording using Sequenced Language Scale for Infants (SELSI; Kim, 2002), a language assessment tool based on parent report. Results from three infants, 2 in the preverbal group and 1 in the multiword group, had signs of language delay. We offered them an opportunity to see a speech pathologist for a proper testing if they wished. Based on advice from experts that the assessment for preverbal infants is prone to error simply because there are too few questions to assess their linguistic ability, we decided not to exclude these children from analyses. The information on the child participants’ age and sex is presented in Table 1.

**Table 1:** Demographics of the infant participants.

Group	Age	Sex
Age 0 (preverbal)	0;6.3–0;9.24 (*M* = 8 months, *SD* = 40 days)	8 Male, 4 Female
Age 1 (early speech)	0;11.14–1;4.4 (*M* = 13 months, *SD* = 41 days)	6 Male, 6 Female
Age 2 (multi-word)	2;1.14–2;6.25 (*M* = 27 months, *SD* = 47 days)	8 Male, 4 Female


Data were collected at a mock apartment laboratory in Seoul National University. The apartment consisted of a foyer, a living room, a bedroom, and a kitchen, though mothers and children played mostly in the living room. Toys and books were available in the living room. The infant-mother dyads had 40 min of free-play followed by a 10 min session to elicit adult-directed speech. During the free-play session, the mothers were told to play and interact with their children as they would usually do at home. The dyads were left alone during the free-play.

Both the mother and the child wore vests fitted with a uni-directional clip-on microphone (Edutige ETM-008) with a signal-to-noise ratio of 69 dB and a frequency response of 100 to 12 KHz. It was plugged into a SONY digital recorder, which was put in the pocket on the vest, and the recording was sampled at 44.1 KHz with 16 bits per sample in a linear PCM format. The two channels were manually synchronized using the hand-clap at the beginning of the recording session as a cue and merged into a single file afterward.

After eliminating 1 recording from the Age 1 group due to the child’s excessive crying, interactions of the 35 dyads were transcribed following the CHAT transcription format (MacWhinney, 2000). A total of 32,011 utterances were transcribed including 24,684 from the mothers.

### Target Words and Coding

The transcribers were instructed to mark lengthened syllables and word play as they transcribed. Following the CHAT transcription format (MacWhinney, 2000), lengthened syllables were marked with a colon *(:)*, and word play was tagged with *@wp*. A word was tagged as containing word play when its prosodic features were significantly modified (e.g., unusually low or high pitch, unusually short or long duration) and thus differed from usual conversational speech. Typical examples of word play include rhythmical utterances and chanting (e.g., *c’akc’ak’uŋ c’akc’ak’uŋ* “clap clap”), similar to the patty cake game in Western culture, and cases in which mothers change their voice to imitate the sound of an animal or an object. We then created a list of ideophones based on the frequency list of all words contained in the transcripts. An undergraduate research assistant generated a preliminary list based on inspection of every word in the frequency list in view of the dictionary definition of ideophones, and the first author refined the list to produce the final list of ideophones. Based on this list, the ideophone word tokens in the transcripts were annotated with the *@o* tag using a custom script.

**FIGURE 1:**
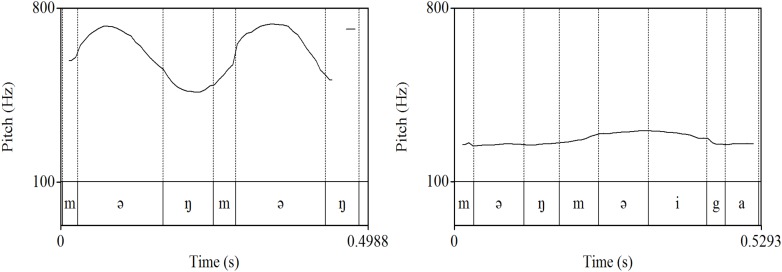
Pitch contours of *məŋməŋ* “woof woof” (left) and *məŋməŋ-i-ka* “woof woof-CITATION-NOM” (right) produced by the same speaker.

It should be noted that some words belonged to more than one of the three categories of expressive lengthening, ideophones, and word play. Different types of target words examined in this study are provided in Table 2. It was expected that many ideophonic words are also judged to involve word play, as in *məŋməŋ* “woof woof” (type a), as the mothers produced them playfully, with substantially modified word duration and pitch measures when they mimicked sounds of an animal or an object, or expressed the dynamic nature of actions. However, some ideophones were not regarded as word play (type b), e.g., when an ideophonic expression was used as a referring term. Note in examples (a) and (b) that the same sequence of segments could be realized with very different prosody depending on whether the lexical item was produced with word play or not (see Figure 1). Some ideophonic words with word play were also accompanied by lengthening (type c), e.g., *ja:oŋ* “meow.” Also, as mentioned earlier, there were instances of word play that were not categorized as ideophones as in (e), e.g., chanting with non-words, since they were produced with the sole purpose of amusing the child without any specific meaning or reference to any target objects.

**Table 2:** Types of target words.

	Expressive lengthening	Ideophones	Word play	Example	# of tokens (types)
a		√	√	*m əŋməŋ* “woof woof”	2958 (782)
b		√		*m əŋməŋ-i-ka* “woof woof-CITATION-NOM”	745 (276)
c	√	√	√	*ja::o ŋ* “meow”	1391 (342)
d	√			*k^h^ə::talan sakwaka* “huge apple-NOM”	124 (55)
e			√	*t∔ t∔ t∔ t∔ t∔ t∔ t∔ t∔ t∔ p’jo ŋ*	882 (397)


We calculated the ratio of word play in ideophonic and non-ideophonic words by dividing the number of (non-)ideophonic words marked as word play by the total number of (non-)ideophonic words. It was found that the majority of ideophones were produced with word play, whereas only a few non-ideophonic words were produced as such. Specifically, about 73.9 percent of ideophonic words featured word play in type frequency and 79.9 percent in token frequency. In comparison, non-ideophonic words were produced with word play in only about 4.0 percent in type frequency and 1.2 percent in token frequency. Therefore, ideophonic words appeared to be much more frequently produced with enhanced prosody compared with non-ideophonic words. We investigate this prediction based on the large overlap between ideophones and word play more systematically with acoustic data, as described later.

### Analysis of Frequency and Acoustic Measures

We extracted all utterances that contained the target words for ideophones, expressive lengthening, and word play based on this initial coding. We then made some refinements on the output of expressive lengthening to conduct the frequency and acoustic analyses. For the frequency analysis, we eliminated all words with the lengthening on the final syllable of the word. The purpose of this cleanup was to exclude tokens with exaggerated word-final lengthening, which is often due to factors other than augmenting the scalable semantic property of the word (e.g., *c^h^ulpal::* “start,” or “let’s go”).

For the acoustic analysis, we first extracted all ideophones that accompanied word play, i.e., all words with the @o@wp tag, and all words with expressive lengthening, i.e., words tagged with colon (:), and sent them to Praat. We then refined the list by a manual and aural inspection since we wanted to include tokens with word-final lengthening that actually reflected the enhanced sound-meaning correspondence, as in monosyllabic adjectives (e.g., *k^h^in::* “big”) or certain instances of ideophones (e.g., *jaoŋ::* “meow”), and also to filter out tokens with word-medial lengthening that were irrelevant to enhancing the relation between sound and meaning (e.g., *catoŋ::c^h^a* “car”).

The prosodic saliency of these sound symbolic words was compared with non-iconic conventional words with regard to duration, mean pitch, maximum pitch, and pitch range of a word. While Laing et al. (2016) compared acoustic features of onomatopoeic words (e.g., *woof woof*) with those of corresponding conventional words (e.g., *dog*), we decided to use randomly chosen non-iconic words for comparison, since there were quite a lot of cases in which ideophonic words did not have a non-ideophonic counterpart. In order to select the conventional words to be used for comparison, we first generated a set of automatically aligned text and the acoustic data by using Prosodylab, a forced-alignment toolkit (Gorman et al., 2011). We then randomized the order of the words in the entire transcript for each mother using a Praat (Boersma and Weenink, 2016) script. Finally, we selected the first 50 to 60 words in each mother’s speech from the first few minutes of the randomized speech, manually eliminating sound symbolic words.

Considering that duration is sensitive to speaking rate, we tested whether any observed change in the duration of sound symbolic words in different age groups is an artifact of the overall speaking rate of mothers’ speech, which changes as a function of child age. Speaking rates of the utterances obtained from mothers’ CDS were calculated using a Praat script (De Jong and Wempe, 2009). For a subset of the utterances, speaking rates were calculated based on a manual syllable count in order to test the reliability of the automatic calculation using the script. Since speaking rate is regulated by the number of words contained in an utterance, we analyzed only utterances containing equal numbers of words. We then selected all utterances containing seven words to be coded for number of syllables.

### Statistical Analysis

For the analysis of the frequency, we conducted a multiple linear regression analysis with the RATIO of sound symbolic words as the dependent variable and AGE (3 levels; 0, 1, 2), GENDER of children (2 levels; female, male), and WORD TYPE (3 levels; ideophones, expressive lengthening, and word play) as fixed effects. Levels underlined in each variable indicate the reference level. For the analysis of speaking rate, we conducted a Pearson correlation analysis to obtain the correlation between speaking rates automatically calculated using the script and those based on manual syllable count. For statistical tests of the acoustic analysis, four mixed effects linear regression models were established, using the *lmer* function from the lmerTest (Kuznetsova et al., 2017) in R (R Core Team, 2016), with DURATION, MEAN PITCH, PITCH RANGE, and MAXIMUM PITCH as dependent variables in each analysis. In all the models, WORD TYPE (3 levels: ordinary, expressive lengthening, ideophones), AGE (3 levels: 0, 1, 2), GENDER of children (2 levels: female, male), and WORD TYPE × AGE interaction were included as fixed effects. Given the idiosyncratic variation due to differences between individual mothers and also between different tokens of the same sound symbolic words, we included SPEAKER and ITEM as random factors.

## Results

### Frequency of Sound Symbolic Words in Mothers’ Speech

In order to investigate change in the frequency of sound symbolic words in mothers’ speech as a function of children’s age, we calculated the ratio of the target words in each mother’s speech by dividing the number of words marked as having lengthened syllables, ideophones, and word play by the total number of words.

**FIGURE 2:**
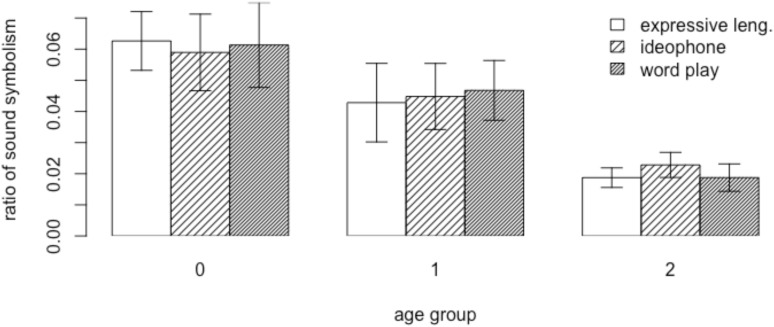
The ratio of sound symbolic words in mothers’ speech.

We constructed a multiple linear regression model with the RATIO of sound symbolic words as the dependent variable and the AGE, GENDER of children, and WORD TYPE as fixed factors. Partial F-tests comparing models with and without the interaction terms among the three fixed effects found no statistically significant interactions (all *p*-values > 0.05). The results of the regression indicated that AGE explained 23% of the variance [*R*^2^ = 0.23, *F*(5, 99) = 5.9, *p* < 0.001]. It was found that the ratio of sound symbolic words in the preverbal group (AGE 0) was significantly greater (β = 0.02, *p* < 0.05), whereas the ratio in the multiword group (AGE 2) was significantly smaller (β = -0.025, *p* < 0.001) than the ratio of those words in the early speech group (AGE 1) (see Figure 2). GENDER or WORD TYPE had no significant effect.

Thus, mothers of children at the early speech stage used sound symbolic words less frequently (*M* = 0.045, *SD* = 0.35) than mothers of infants that are preverbal (*M* = 0.061, *SD* = 0.04), and, in turn, mothers of children at the multiword stage used sound symbolic words in a smaller proportion (*M* = 0.02, *SD* = 0.13) than mothers of children just beginning to produce speech. Overall, these findings suggest that mothers adapt their speech to match their child’s changing linguistic ability, by reducing the amount of sound symbolic words in the input and increasing “conventional” words over time.

### Acoustic Saliency of Sound Symbolic Words

We investigated whether the two categories of sound symbolic words examined in this study, i.e., expressive lengthening and ideophones, were acoustically more salient than non-iconic conventional words in duration, mean pitch, pitch range, and maximum pitch. Recall that one of the purposes in our analysis of ideophones was to confirm our informal observation of the saliency of these words based on a systematic acoustic study. Our critical interest was to investigate whether such saliency becomes weaker as a function of child age, with decreased durational or pitch values as children grow older. Target words of ideophone, expressive lengthening, and word play were selected based on the procedures described above. Initially, a total of 2,958 ideophonic word tokens produced with word play and 1,297 word tokens produced with expressive lengthening were found. After excluding tokens that were not suitable for acoustic analysis due to background noise or overlapping speech, 1,460 ideophonic words involving word play and 641 instances of expressive lengthening were compared with a randomly generated sample of 1,885 ordinary words in terms of duration and pitch measures.

#### Duration

**FIGURE 3:**
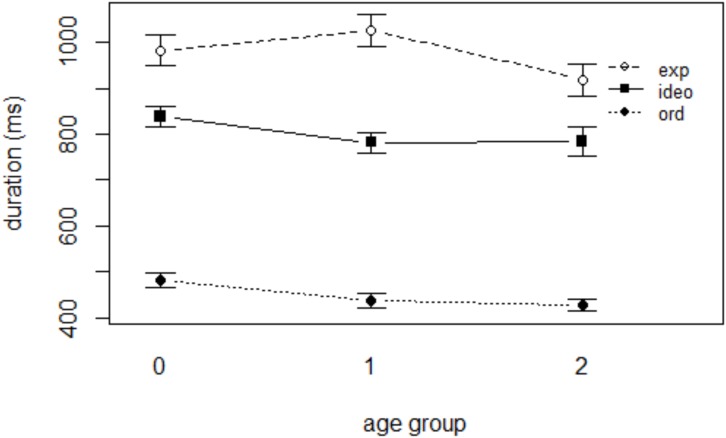
Duration of the three word categories in each age group.

The results of mixed effects linear regression analysis with DURATION as the dependent variable are presented in Table 3. The effect of WORD TYPE was significant; both expressive lengthening and ideophones were significantly longer than ordinary words (see Figure 3; all *p*-values < 0.001). The coefficients indicate that words that underwent expressive lengthening, which should by definition be long in duration, were 474 ms longer than ordinary words, which had a mean duration of 526 ms, whereas ideophonic words were longer than ordinary words by 363 ms. The effect of AGE or GENDER was not significant (*p*’s > 0.05). None of the WORD TYPE × AGE interactions was significant (*p*’s > 0.05), indicating that the difference in duration between ordinary words and sound symbolic words did not vary significantly with child age.

**Table 3:** Mixed effects linear regression results (duration).

	Estimate	Std. Error	*t*-value	Pr (>|t|)
(Intercept)	526.169	27.394	19.207	<0.001
WORD TYPE (exp)	474.424	63.014	7.529	<0.001
WORD TYPE (ideo)	363.125	47.128	7.705	<0.001
AGE (1)	–18.390	28.747	–0.640	0.527
AGE (2)	–32.223	27.940	–1.153	0.258
GENDER (male)	8.607	22.901	0.376	0.710
WORD TYPE (exp):AGE (1)	31.816	86.667	0.367	0.716
WORD TYPE (ideo):AGE (1)	–35.451	61.671	–0.575	0.570
WORD TYPE (exp):AGE (2)	–101.252	87.281	–1.160	0.254
WORD TYPE (ideo):AGE (2)	–71.643	62.775	–1.141	0.262


**FIGURE 4:**
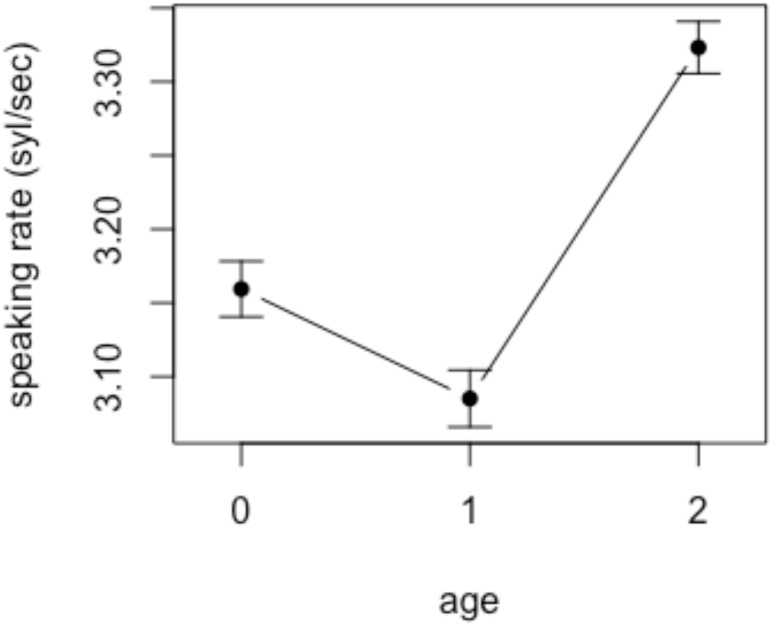
Speaking rate of mothers’ speech in each age group.

To inspect the possibility of these results being affected by age-dependent variation in speaking rates, we investigated mothers’ speaking rates as a function of child age using a Praat script (De Jong and Wempe, 2009). We extracted all utterances produced by the mothers, and eliminated the ones that contained the following; non-speech sounds such as laughing; unintelligible speech due to poor signal quality; playful vocalization including singing and role-playing. Out of the total of 24,684 utterances produced by the mothers, 21,128 were analyzed. The number of utterances included in the analysis from each age group was as follows: 7,916 (preverbal), 8,152 (early speech), and 8,616 (multiword). It was found that mothers’ speaking rate, defined as number of syllables per second, slows down around the time the children begin to speak but rapidly accelerates once the children begin producing words (see Figure 4), which is consistent with the findings with English-speaking mothers (Ko, 2012). Though not reported here in detail, individual variation among mothers’ speaking rate was greatest when the child was preverbal, again consistent with the findings in Ko (2012). A mixed effects linear regression model was constructed with AGE (3 levels: 0, 1, and 2) as a fixed effect and SPEAKING RATE as a dependent variable. To account for the idiosyncratic discrepancies among individual speakers and repeated utterances, SPEAKER and UTTERANCE were included as random intercepts. The results revealed that the difference in the mothers’ speaking rate between age 0 and 2 was significant (*p* < 0.05), whereas mothers of children in age groups 0 and 1 did not differ significantly in their speaking rate (*p* > 0.05).

In order to check the reliability of the automated measure of speaking rate reported above, we conducted a manual analysis of speaking rate on a subset of the utterances. Given the effect of utterance length on word duration, we selected a total of 580 utterances containing an arbitrarily chosen number of words, i.e., seven, in the mother’s speech. As we listened to each utterance, we excluded those containing overlapping speech, laughs, disfluencies, and playful vocalizations. The number of utterances thus included in the analysis was 272. When counting the number of syllables, care was taken to count the syllables as actually produced by the speakers rather than make reference to the transcripts. It was found that the correlation between the number of syllables counted manually and automatically was moderately high (*r* = 0.72, *p* < 0.001).

In sum, the results of the duration analyses show that while the overall speaking rate increased with child age, there was no significant effect of age on the durations of target words in this study. These results, put together, indicate that mothers do not shorten the duration of sound symbolic words up to age 2 despite their overall speaking rate getting faster.

#### Pitch Measures

**FIGURE 5:**
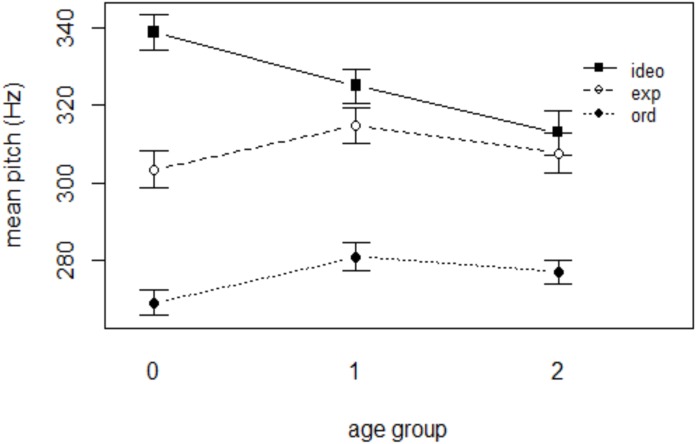
Mean pitch of the three word categories in each age group.

Table 4 shows the results of the mixed effects linear regression analysis for mean pitch. Ordinary words had a mean pitch of 272 Hz on average. When a word included expressive lengthening, the mean pitch of the word was significantly higher than ordinary words (*p* < 0.05, Figure 5) and ideophonic words were also significantly higher in pitch than ordinary words (*p* < 0.001). Words that underwent expressive lengthening were 20 Hz higher in mean pitch than ordinary words, whereas ideophonic words were 54 Hz higher than ordinary words. As was the case in the duration model, the effect of AGE and GENDER was not significant (*p*’s > 0.05). Interestingly, there was a significant WORD TYPE (ideo) ×AGE (2) interaction (*p* < 0.05), indicating that the acoustic saliency (i.e., higher pitch) of ideophones became weaker with child age. In contrast, however, no such effect was found for the interaction between expressive lengthening and any age of the groups. The results of the interaction between WORD TYPE and AGE, taken together, suggest that mothers maintain the acoustic saliency for expressive lengthening but weaken the prominence for ideophones when the child reaches a certain age.

**Table 4:** Mixed effects linear regression results (mean pitch).

	Estimate	Std. Error	*t*-value	Pr (>|t|)
(Intercept)	272.482	9.827	27.727	<0.001
WORD TYPE (exp)	20.603	8.349	2.468	0.017
WORD TYPE (ideo)	54.611	8.109	6.735	<0.001
AGE (1)	9.299	12.010	0.774	0.444
AGE (2)	6.357	11.813	0.538	0.594
GENDER (male)	–5.079	7.659	–0.663	0.512
WORD TYPE (exp):AGE (1)	–9.638	11.246	–0.857	0.397
WORD TYPE (ideo):AGE (1)	–12.710	10.902	–1.166	0.253
WORD TYPE (exp):AGE (2)	–2.505	11.923	–0.210	0.834
WORD TYPE (ideo):AGE (2)	–23.818	11.256	–2.116	0.041


As for pitch range and maximum pitch, the results of regression analyses were similar for these two variables. The difference between ordinary words and ideophonic words was significant for both pitch range (*p* < 0.01) and maximum pitch (*p* < 0.001). However, the difference in these two pitch measures between ordinary words and words with lengthened syllables was not significant (*p*’s > 0.05). The effect of AGE and GENDER was not significant (*p*’s > 0.05). None of the WORD TYPE × AGE interaction terms was significant (*p*’s > 0.05), suggesting that the phonetic prominence of sound symbolic words persisted throughout the age range of children investigated in this study.

In a separate regression model for pitch range, we converted f0 range in Hertz to semitone. The significance of each fixed factor remained the same; the difference in pitch range measured in semitones between ideophones and ordinary words was significant (*p* < 0.05), whereas no other factor was shown to be significant.

## Discussion

This study examined whether the frequency of sound symbolic words in Korean child-directed speech and the acoustic saliency of these iconic words were related to child age. The frequency analysis showed that the ratio of sound symbolic words in mothers’ speech was higher when children were younger, consistent with findings of previous studies (e.g., Saji and Imai, 2013). The age effect we found might relate to linguistic advances rather than age, but it was hard to investigate directly this possibility since the majority of our child participants were still younger than 14 months; thus measures such as vocabulary size or MLU were not easily available. An acoustic analysis also showed that sound symbolic words were more acoustically salient than non-iconic conventional words in duration and pitch measures, confirming the findings by Laing et al. (2016).

Beyond such confirmatory analyses, however, this study set out to investigate any possible modulation of such acoustic saliency as a function of child age in two different types of sound symbolic words. To our knowledge, ours is the first attempt to investigate the interaction between word type and age of children in acoustic measures relating to sound symbolism. The degree to which mean pitch was raised in ideophonic words was larger in mothers’ speech directed to the children of the youngest group compared with that of the oldest group. When children are younger and thus are still mastering the ability to associate linguistic form with meaning, mothers might provide clearer cues, i.e., an additional enhancement of mean pitch, to help their children recognize that words refer to entities in the world. As children’s linguistic ability improves, however, mothers might find it less necessary to provide such redundant information. More generally, however, we did not find a robust age effect on the acoustic saliency of sound symbolic words, at least within the age range of children investigated in this study, aside from the mean pitch difference between ideophones and ordinary words just discussed. For duration, pitch range, and maximum pitch, the acoustic saliency of sound symbolic target words was constant across children’s age. Therefore, we can infer that children at the age of 2, the oldest age group examined in this study, might still benefit from the enhanced prosody of the iconic words, particularly as regards adjectives and adverbs, and that mothers accordingly continue to provide these redundant acoustic cues in their speech.

To understand this somewhat unexpected finding, in particular the lack of interaction between the WORD TYPE (expressive lengthening) and AGE in all acoustic measures, it might be enlightening to consider the properties of the words undergoing expressive lengthening and the developmental schedule of children’s word learning. Recall that most words undergoing expressive lengthening in Korean are adjectives or adverbs of scalar property (Ko, 2017). Previous research has shown that relative scalar properties of adjectives will be understood by children quite late in the period of most intensive word-learning (Clark, 1970; Gitterman and Johnston, 1983; Smith et al., 1986, 1988) and their findings indicate that even three-year-olds identify particular attributes such as *high* and *low* as two polar extremes, not realizing that they exist on a continuum. Clark (1972) also argued that 4- and 5-year-olds first produce two adjective opposites that are later subdivided into a full continuum. Even the oldest group of children in this study then are likely to not have developed their ability to discern the relational nature of certain attributes. We could, therefore, infer that they might still benefit from the presentation of the scalable adjectives with acoustic prominence. Findings from previous research hint that mothers might convey meaning with different levels of scalar properties when they produce expressive lengthening. Specifically, Kawahara and Braver (2013) report that Japanese speakers make fine-grained distinctions in vowel duration, as many as six levels, to encode different levels of emphasis and that most participants showed a correlation between vowel duration and the scalar property of the target adjective. It is, therefore, plausible that exposure to expressive lengthening might be beneficial for the listener to associate a scalar property with the meaning of the adjective in question. This explanation, however, would have to be confirmed by directly testing the benefit of expressive lengthening in learning scalar adjectives and the degree of its universality.

Let us now consider the divergence between the ideophones and expressive lengthening with regard to their interaction with age in mean pitch. Despite the overall lack of an age effect on the acoustic saliency of sound symbolism, we found that the degree to which mean pitch was raised in ideophones decreased with child age. Why would mothers treat ideophones in a different way from expressive lengthening? It has been pointed out that sound symbolic words might *not* be useful in certain aspects of word learning, such as making fine-grained distinctions among similar concepts. Some studies have shown that sound symbolism promotes word learning for the basic level category labels, but does not carry any advantage for learning individual words (Monaghan et al., 2011, 2012). These studies suggest that sound symbolism can facilitate word-category mappings (e.g., some phonological features associated with actions and others with objects; some segments with rounded objects and others with angular objects), but they inhibit individual word-referent mappings within the same category. As Imai and Kita (2014) pointed out, during the earliest stages of language learning, infants rarely face the situation in which they have to make fine distinctions among similar referents; they only need to learn basic category labels (*bird* rather than *robin*). As infants become experienced learners, however, finer discrimination among similar concepts may be needed. Older children thus need to learn more “conventional” words in which the arbitrary relationship between sound and lexical meaning facilitates the acquisition of specific word meanings. In contrast, children learn to discriminate the fine details of scalar properties and their conversational implicatures of adjectives only later in learning. Therefore, unlike the case of ideophones, mothers might be providing acoustic salience for these words undergoing expressive lengthening in all relevant measures, not just in the domain of duration, throughout the developmental periods we examined.

The present study is one of the few investigating the properties of CDS in Korean, a language where sound symbolic words occupy a special status in the lexicon not only in terms of relative frequency but also because they form a lexical stratum for phonological rule applications. This study, therefore, makes an important contribution in providing confirmatory results of the findings made in previous research based on typologically different data. Further, this study extends the scope of types of sound symbolism investigated in caregiver speech. While previous studies mostly focused on the use of onomatopoeia or mimetics and found that the frequency of those words in mothers’ speech decreased as a function of the child’s age (e.g., Saji and Imai, 2013) and that they were phonetically more prominent than conventional words (Laing et al., 2016), the present study found similar trends in expressive lengthening as well. It would be interesting to compare the effect of age on acoustic measures of expressive lengthening in English-speaking mothers with the findings of our study.

When a child with a small lexicon hears a word, she may find it challenging to figure out which part of the world the word refers to. Parents can provide a variety of cues to help children unambiguously associate the word with the referent, e.g., by pointing to the object (Butterworth, 2003) or naming the object while the child is attending to it (temporal synchrony; Gogate et al., 2006). However, as suggested in Imai and Kita (2014), because pointing and temporal co-occurrence can only help children attend to here-and-now associations between the word and the referent, these strategies cannot aid children in using the word in a new situation. To use a word beyond a specific here-and-now context, children need to identify the invariance in the word meaning across various contexts in which the word appears. The direct link between phonological form and word meaning found in sound symbolism can help children extract the invariance in the word meaning, enabling them to generalize the use of a word to new contexts. Therefore, sound symbolic words in child-directed speech can facilitate the infants’ acquisition of word meanings.

Infants’ sensitivity to cross-modal and multimodal cues is also supported by an increasing number of studies. Although infants’ perceptual sensitivity to expressive lengthening itself has not been examined in previous studies, the so-called *bouba-kiki* effect has been observed in young children where they mapped *bouba* with rounded objects and *kiki* with angular ones (Maurer et al., 2006; Asano et al., 2015). It was also found that infants integrate visual and audio cues for perception, known as the McGurk effect (Kuhl and Meltzoff, 1982; Rosenblum et al., 1997). Moreover, there is evidence showing that mothers’ touches are aligned with their speech in a systematic way, suggesting that tactile cues could assist infant word segmentation and word learning (Abu-Zhaya and Seidl, 2017). As such, infants seem to use multimodal cues in language learning in various perceptual domains such as visual-auditory and tactile-auditory, thus benefiting from sound symbolism which in most cases inherently encodes cross-modal information (see Choi et al., 2018 for a recent review on the effect of early multisensory signals on infants’ language ability). A future study directly testing the benefits of the acoustic saliency encoded in expressive lengthening for infants’ word learning would strengthen our understanding of word learning mechanisms in children.

## Conclusion

We investigated the use of sound symbolism in speech directed to children by Korean mothers, focusing on the two different categories of ideophones and expressive lengthening. Our study differs from previous research in that we investigated the effect of age on acoustic variables of prominence. The main finding of our studies is that mothers maintain acoustic saliency for sound symbolic words until later in development, although they somewhat weaken the prominence for ideophones when the child reaches a certain age. Therefore, we can infer that children at age 2, the oldest age group examined in this study, may still be taking advantage of the enhanced prosody of the iconic words and that mothers continue to provide these redundant acoustic cues in their speech. In addition, we found that Korean mothers use expressive lengthening, ideophones, and word play more frequently when talking to younger infants, confirming the findings of similar previous research in other languages.

The less frequent use of sound symbolism and decreased acoustic saliency of ideophones in Korean mothers’ speech to older infants are consistent with the prediction of the soundsymbolism bootstrapping hypothesis (Imai and Kita, 2014; Perry et al., 2017); mothers extensively use sound symbolic words with exaggerated prosody, particularly for ideophones, in the initial stages of learning to help the child learn new words more easily, but they gradually replace those words with more conventional ones (as in adult speech) so that their speech is “just challenging enough” (Zimmerman et al., 2009) to the child learners. Further, the decrease of pitch in ideophones in contrast to the overall lack of age effect in all acoustic measures in expressive lengthening suggests that mothers might move toward more sophisticated forms of interaction with their children, fine-tuning their speech in a way that matches the children’s linguistic abilities. This fine-tuning hypothesis will need to be further tested by future studies focusing on individual variability in the child’s linguistic advancement and the mother’s use of sound symbolism and other features of child-directed speech.

## Ethics Statement

This study was carried out in accordance with the ethical standards of the Institutional Review Board of Seoul National University. The protocol was approved by the Institutional Review Board of Seoul National University (Approval No. 1602/001-007). All participating mothers gave written informed consent in accordance with the Declaration of Helsinki.

## Author Contributions

EK conceived and directed the study. JJ carried out the implementation and reviewed the literature. All authors analyzed the data, interpreted the results, and wrote up the paper.

## Conflict of Interest Statement

The authors declare that the research was conducted in the absence of any commercial or financial relationships that could be construed as a potential conflict of interest.
